# 4‐Octyl Itaconate Suppresses Virulence of Multidrug‐Resistant Avian Pathogenic *Escherichia coli* via Cysteine Alkylation of LuxS, Icl, and OmpA

**DOI:** 10.1155/tbed/5663447

**Published:** 2026-10-25

**Authors:** Saqib Nawaz, Xiaoxian Liu, Haiyang Wang, Yaqin Tian, Jingwen Zhang, Wei Jiang, Xiangan Han, Song Xiangjun

**Affiliations:** ^1^ Anhui Province Key Laboratory of Veterinary Pathobiology and Disease Control College of Animal Science and Technology Anhui Agricultural University Hefei 230036 China ahau.edu.cn; ^2^ Joint Research Center for Food Nutrition and Health of IHM Anhui Agricultural University Hefei China ahau.edu.cn; ^3^ Anhui Province Engineering Laboratory for Animal Food Quality and Bio-safety College of Animal Science and Technology Anhui Agricultural University Hefei 230036 China ahau.edu.cn; ^4^ Shanghai Veterinary Research Institute Chinese Academy of Agricultural Sciences 518 Ziyue Road, Minhang Shanghai 200241 China caas.cn; ^5^ Key Laboratory of Agri-products Quality and Biosafety Anhui Agricultural University Ministry of Education Hefei 230036 China ahau.edu.cn

**Keywords:** 4-octyl itaconate, antimicrobial alternative, APEC, avian colibacillosis, cysteine alkylation, metabolic disruption, poultry gut microbiota safety, virulence attenuation

## Abstract

Avian pathogenic *Escherichia coli* (APEC) is the cause of avian colibacillosis, which leads to huge economic losses in poultry production worldwide, and presents significant zoonotic antimicrobial resistance (AMR) risk. Cell‐permeable itaconate derivatives exhibit dual antibacterial and immunomodulatory properties; however, they have not been systematically evaluated against APEC, and their underlying molecular mechanisms remain poorly defined for this pathogen. In the present study, we systematically explored the anti‐APEC therapeutic potential and mechanism of action of 4‐octyl itaconate (4‐OI) using an integrated approach of in vitro phenotypic, ex vivo macrophage, biochemical genetic, and dual in vivo infection models. In vitro phenotypic assays revealed that 400 μM 4‐OI significantly inhibited the growth of the clinical isolate O78‐type APEC94 and increased its susceptibility to physiological osmotic and acid–base stress (pH 5–7) and decreased the biofilm formation by 64%, swarming motility by 57%, macrophage adhesion by 42%, and cellular invasion by 35%. Ex vivo, 4‐OI showed a dose‐dependent inhibitory effect on the intracellular survival of APEC in RAW264.7 macrophages, with equivalent efficacy to tetracycline, and without any host cell cytotoxicity. Mechanistic qPCR confirmed that 4‐OI inhibits the transcription of five key virulence genes (*fimC*, *luxS*, *wza*, *iroN*, and *menB*) by 2.4–5.3‐fold. Further biochemical pull‐down, LC‐MS/MS, site‐directed cysteine mutation, and gene knockout/overexpression assays demonstrated that 4‐OI directly alkylates the conserved cysteine residues of LuxS, isocitrate lyase (Icl), and OmpA, thereby simultaneously disrupting bacterial quorum sensing, glyoxylate shunt metabolism, and outer membrane integrity. The virulence‐attenuating effect of 4‐OI was confirmed by metabolite complementation assays, which showed that glyoxylate and menaquinone biosynthesis are defective. In vivo therapeutic evaluation showed that 50 mg/kg 4‐OI could significantly suppress APEC‐induced mouse mortality from 87.5% to 12.5%, reduce bacterial loads in serum and all visceral tissues, and downregulated proinflammatory cytokines (IL‐1β and TNF‐α) to relieve over‐immune pathological damage. 50 mg/kg 4‐OI increased the survival of chicken embryos to 86.7% and reduced the bacterial loads in the allantoic fluids by 83% in poultry‐relevant specific pathogen‐free (SPF) chicken embryo models. Moreover, 4‐OI exhibited antibiofilm and antiadhesion activity against various epidemic APEC serotypes and clinical multidrug‐resistant isolates with no effect on the diversity or composition of chicken cecal microbiota. Collectively, our findings support 4‐OI as a safe alternative to conventional antibiotics for treating avian colibacillosis, acting via cysteine alkylation to repress multiple virulence‐ and metabolic‐related targets. This work establishes a multifaceted preclinical proof‐of‐concept dataset supporting further development of 4‐OI as an anti‐multi‐drug resistance (MDR) candidate agent; additional farm‐relevant in vivo trials will be required prior to real‐world application for safeguarding poultry health and global food safety.

**Summary**

• About 400 μM 4‐octyl itaconate (4‐OI) substantially suppresses core virulence phenotypes in vitro: biofilm formation (−64%), macrophage adhesion (−42%), swarming motility (−57%), and cellular invasion (−35%), alongside impaired growth and increased susceptibility to osmotic‐ and acid‐stress.
• 4‐OI is a cysteine alkylator that covalently modifies the conserved cysteine residues of LuxS, isocitrate lyase (Icl), and OmpA and at the same time inhibits quorum sensing, glyoxylate metabolism, and outer membrane virulence pathways. They are essential functional targets for 4‐OI activity, according to genetic knockout and cysteine point mutagenesis.
• 4‐OI dose‐dependently reduces intracellular avian pathogenic *Escherichia coli* (APEC) survival within macrophages without host‐cell toxicity, exhibiting bacteriostatic activity comparable to tetracycline while limiting macrophage inflammatory injury via the suppression of TNF‐α/IL‐1β production.
• In the mouse model of APEC‐induced mortality, 50 mg/kg 4‐OI reduces mouse mortality from 87.5% to 12.5%, increases survival of specific pathogen‐free (SPF) chicken embryos to 86.7%, and dramatically decreases loads of bacteria in the systemic and mucosal tissues.
• Broad‐spectrum activity across clinically relevant APEC serotypes (O1, O2, O18, O78, O88, and O145) and clinical multidrug‐resistant APEC field isolates; consistent efficacy against pre‐existing MDR isolates indicates absence of preformed cross‐resistance to conventional antibiotics.
• For poultry‐industry applications, 4‐OI represents a safe antibiotic‐alternative feed additive candidate to mitigate antimicrobial resistance (AMR)‐associated losses without altering α‐/β‐diversity or taxonomic composition of the chicken cecal microbiota.

## 1. Introduction

Avian colibacillosis is primarily caused by avian pathogenic *Escherichia coli* (APEC), an encapsulated, flagellated, and fimbriated Gram‐negative bacterium. This highly contagious disease can produce systemic inflammatory conditions such as airsacculitis, perihepatitis, and pericarditis, resulting in high morbidity and mortality rates in commercial poultry production and significant economic losses to the poultry industry throughout the world [1, 2]. APEC represents a critical zoonotic pathogen. Globally disseminating APEC strains frequently acquire multi‐drug resistance (MDR), rendering conventional antibiotics progressively less effective and raising substantial public‐health risks through potential cross‐species transfer of antimicrobial‐resistance (AMR) determinants [3]. APEC pathogenicity is a coordinated array of virulence properties; these include a strong ability to form a biofilm, survival in oxidative, acidic, and osmotic stress conditions, and to efficient attachment and invasion of immune cells as well as survival within them. These phenotypes allow APEC to escape host immune surveillance, colonize several mucosal and systemic tissues, and cause persistent infections [4–6]. The existing alternative control measures such as vaccines, probiotics, and bacteriophages have limitations, such as serotype‐specific immunity for vaccines, requirement of stable intestinal colonization for probiotics, and narrow host range for phages [7]. The need for broad‐spectrum, safe antivirulence small molecules that can replace the overused antibiotics is dire [8, 9].

Itaconate is a canonical tricarboxylic acid (TCA) cycle intermediate that has dual and broad antibacterial and immunomodulatory effects [10]. Itaconate is a native metabolite that disrupts isocitrate lyase (Icl), a rate‐limiting enzyme of the glyoxylate shunt, which allows pathogen survival in nutrient‐restricted host intracellular environments. Indigenous itaconate, however, is highly polar and has poor cell permeability and, therefore, is poorly effective in vivo for therapeutic purposes [11]. The esterified itaconate derivative 4‐octyl itaconate (4‐OI) is metabolized intracellularly to generate bioactive itaconate, possesses electrophilic moieties capable of covalent reaction with nucleophilic cysteine thiol groups, and is readily taken up into bacterial and mammalian cells [11, 12].

Previous studies have confirmed the activity of 4‐OI against nonavian pathogens such as *Salmonella enterica*, *Mycobacterium tuberculosis*, and *Legionella pneumophila* [13, 14]. In fact, all current studies have neglected to systematically examine 4‐OI in relation to APEC, and no previous study has found direct targets of 4‐OI via binding to bacterial proteins, nor has the mode of bacterial protein alkylation by 4‐OI been proven for Enterobacteriaceae [15, 16]. Our preliminary transcriptomic data showed significant downregulation of APEC virulence genes following 4‐OI treatment, but the phenotypic, metabolic, and in vivo functional effect of this transcriptional repression was not validated [17]. Although initial transcriptomic data are promising, three fundamental research gaps remain in the literature: (1) no systematic phenotypic and therapeutic profiling of 4‐OI against APEC has been performed; (2) the practical feasibility of deploying 4‐OI for APEC control has rarely been evaluated; and (3) critical mechanistic questions remain unresolved, including the identity of direct 4‐OI protein‐binding partners and whether cysteine alkylation mediates antivirulence effects in APEC [13, 18–20]. Prior work has only reported altered virulence‐gene transcription without assessing downstream phenotypes such as bacterial growth, stress resistance, biofilm formation, motility, macrophage intracellular survival, or outcomes in avian infection models. Furthermore, translational safety data supporting 4‐OI as a poultry feed additive are absent; in particular, the impact of 4‐OI on chicken cecal commensal microbiota has not been characterized [17].

Building on these identified research gaps and our preliminary transcriptomic data, we formulated the following core hypothesis: Membrane‐permeable 4‐OI covalently modifies conserved cysteine residues of key APEC virulence and metabolic proteins, simultaneously inhibiting virulence‐gene transcription and disrupting central carbon metabolism. These effects ultimately attenuate APEC pathogenicity while preserving host inflammatory homeostasis without perturbing poultry intestinal commensal bacteria [5]. To test this hypothesis completely, we characterized the inhibitory impact of 4‐OI on the fitness and virulence phenotypes of APEC, identified direct 4‐OI protein‐binding partners by biochemical screening, and confirmed 4‐OI’s core functional inhibitory mechanism through gene knockout, genetic complementation, and cysteine point mutagenesis experiments. We also tested 4‐OI therapeutic activity in the systemic mouse‐infection model and the specific pathogen‐free (SPF) chicken‐embryo avian‐infection model, assessed its activity across diverse APEC serotypes and clinical MDR isolates, and evaluated 4‐OI biocompatibility with macrophages and chicken gut microbiota for potential industrial development as an antibiotic alternative for poultry [17]. This work generates a comprehensive multidimensional dataset for itaconates as a mechanistic and translational tool to combat APEC.

## 2. Materials and Methods

### 2.1. Experimental Materials

#### 2.1.1. Bacterial Strain, Small Molecule, and Biochemical Reagents

The clinical isolate APEC94 serotype (O78) was saved in the laboratory strain library as the primary experimental strain. For consistency check, 4‐OI was bought from two commercial suppliers, Sigma–Aldrich (USA) and Shanghai Lanjun Biotechnology Co., Ltd. (China). Analytical‐grade DMSO, LB broth/agar powder, crystal violet, NaCl, gentamicin, Triton X‐100, and sodium deoxycholate (DOC) were purchased from domestic general chemical suppliers. Cell culture medium (DMEM), fetal bovine serum (FBS), penicillin–streptomycin double antibody (P/S), and MTT powder were obtained from Thermo Fisher Scientific (USA). Molecular kits: Qiagen total RNA extraction kit, TaKaRa reverse transcription kit, SYBR Green qPCR Master Mix, and Luminex 32‐plex mouse cytokine magnetic bead detection kit (Millipore Sigma, USA). Metabolic intervention reagents (PaβN, succinate, malonate, CCCP, and 2‐deoxyglucose) and tetracycline (positive control) were obtained from Sigma–Aldrich (USA). Biotin‐conjugated 4‐OI probe (biotin‐4‐OI) was custom‐synthesized by Shanghai ChemPartner Co., Ltd. Purity >95% was confirmed by HPLC‐HR‐MS. 4‐OI was obtained from two vendors for batch consistency validation: Sigma‐Aldrich (USA, Cat# SML2338, ≥98% purity) and Shanghai Lanjun Biotechnology Co., Ltd. (China, Cat# LJ‐OI‐001, ≥97% purity). Initial phenotypic validation assays (growth curve and biofilm inhibition) were run side‐by‐side for both preparations; no statistically significant differences in APEC inhibitory phenotypes were observed across sources. All main‐study in vitro cellular, biochemical, and animal in vivo experiments were performed using Sigma‐Aldrich 4‐OI; Lanjun‐sourced material was reserved for replicate confirmatory phenotypic assays only.

#### 2.1.2. Cell Line, Laboratory Animals, and SPF Chicken Embryos

The RAW264.7 mouse macrophage cell line was purchased from the American Type Culture Collection (ATCC). The 6‐week‐old male C57BL/6 mice (*n* = 24; total) were provided by Jiangsu Huachuang Sino Pharmaceutical Co., Ltd. (China) and maintained in SPF animal facilities. Ten‐day‐old SPF chicken embryos (45 in total) were obtained from Beijing Merial Vital Laboratory Animal Technology Co., Ltd. (China).

### 2.2. Experimental Protocols

#### 2.2.1. Bacterial Culture and 4‐OI Stock Preparation

APEC94 was cultured aerobically in a LB medium at 37°C with shaking (200 rpm) or statically cultured on LB agar plates. Unless otherwise indicated, mid‐logarithmic‐phase bacteria (OD_600_ = 0.6–0.8) were used for all experiments. 4‐OI powder was dissolved in DMSO to create a 100 mM mother liquor, which was stored at −20°C in the dark and diluted to the desired concentrations (0–400 μM) in culture medium just prior to each experiment. All treatment groups contained DMSO with a final concentration less than 0.1%, and this did not affect bacterial growth or the viability of macrophages. The in vivo therapeutic dose of 50 mg/kg was determined from our previously published mouse gradient administration assays (Section 2.2.6.3), and the in vitro working concentration was established at 400 μM following gradient toxicity and phenotypic screening experiments [21].

#### 2.2.2. In Vitro Phenotypic Assays of APEC94

##### 2.2.2.1. Growth Curve Measurement

APEC94 bacterial suspension was diluted 1:100 in 100 mL of LB medium with 400 μM 4‐OI or an equivalent amount of DMSO solvent control. The cultures were shaken at 180 rpm at 37°C, and the absorbance at 600 nm was measured every 2 h during 24 h of incubation in a Bio‐Rad spectrophotometer to draw the 24 h growth curves. In all experiments, biological triplicates were made. MIC and MBC were determined for APEC94: MIC = 350 μM; MBC > 800 μM for APEC94. Thus, 400 μM represents a bacteriostatic near‐MIC concentration rather than a bactericidal condition for this strain.

##### 2.2.2.2. Osmotic Stress Resistance Assay

Mid‐log APEC94 (OD_600_ = 1.0) bacterial suspension (200 μL) was inoculated into 3 mL of LB medium supplemented with 0.5%, 1%, 2%, or 4% (w/v) NaCl. These gradients were split into solvent controls and 400 μM 4‐OI treatment and then shaken at 37°C for 1 h. A 10‐fold gradient dilution was made with sterile PBS and spread on LB agar, with incubation for 12 h. After 20 μL of the diluent was added onto the medium, the number of colony‐forming units (CFUs) was counted, and the survival rate under osmotic stress was calculated. Three biological replicates were carried out.

##### 2.2.2.3. Acid–Base Stress Tolerance Assay

The LB medium was adjusted to pH 4, 5, 6, 7, 8, 9, and 10, respectively. 400 μM 4‐OI or an equal volume of DMSO vehicle control was added to 5 mL of medium of each pH to each group and incubated at 37°C with shaking for 12 h. OD_600_ was used as a readout for total culture biomass under pH stress conditions; OD turbidity cannot discriminate viable versus nonviable cells. Future assays will complement these measurements with CFU plating for direct viable‐cell enumeration.

##### 2.2.2.4. Biofilm Formation Quantification

A 150 μL LB medium (with/without 400 μM 4‐OI) was placed in each well of a 96‐well plate, and then 15 μL of the mid‐log APEC94 suspension (OD_600_ = 1.0) was added. Biofilms were formed using static incubation at 25°C for 16 h. Static incubation at 25°C was selected because this condition promotes abundant extracellular matrix accumulation and mature biofilm formation for APEC94; 37°C incubation favors planktonic growth and yields reduced mature biofilm biomass for this clinical isolate. Wells were washed with PBS three times, dried at 60°C, stained with 0.1% crystal violet for 20 min, washed, and air‐dried. The crystal violet bound to the biofilm was dissolved in 95% ethanol, and OD_595_ was measured by a microplate reader to estimate the biomass of the biofilm.

##### 2.2.2.5. Swarming Motility Assay

Soft‐agar LB plates supplemented with either 400 μM or 0 μM 4‐OI were prepared. Mid‐log‐phase APEC94 (OD_600_ = 1.0) was spot‐inoculated onto the center of each plate (5 μL) and incubated at 37°C for 12 h. Swarming‐ring diameter was measured to quantify bacterial motility.

#### 2.2.3. Macrophage Cell Culture and Cellular Functional Assays

RAW264.7 macrophages were maintained in culture in complete DMEM (10% FBS + 1% penicillin–streptomycin) at 37°C in a humidified, 5% CO_2_ incubator. The cell density of the cells was determined according to the experimental needs: 1 × 10^5^ cells/well for viability, adhesion, and invasion assays (24/96‐well plates) and 1 × 10^6^ cells/well for itaconate LC‐MS/MS and inflammatory protein western blot (6‐well plates). After overnight adherence, all cell experiments were performed at an 80% confluence.

##### 2.2.3.1. Bacterial Adhesion and Invasion Assays

For the adhesion assay, RAW264.7 cells were infected with APEC94 at MOI = 50, followed by the addition of 400 μM 4‐OI or DMSO at the time of infection. After 2 h incubation, unbound bacteria were washed off with PBS, cells were lysed with 0.2% Triton X‐100, gradient‐diluted, and spread on LB plates for counting of CFU. For the invasion assay, extracellular bacteria were removed after 2 h infection by 1 h treatment with 100 μg/mL gentamicin, and after cell lysis, the number of intracellular invasive bacteria was counted as CFU.

##### 2.2.3.2. Intracellular APEC Survival Kinetics

Macrophages were pretreated with 400 μM 4‐OI or DMSO for 2 h, followed by infection with APEC94 at MOI = 50. 1 h after infection, gentamicin was added to kill the extracellular bacteria. Intracellular bacterial culture was performed at 6, 12, 18, 24, 30, and 36 h postinfection, with blank gentamicin control groups to ensure complete elimination of the extracellular strains.

##### 2.2.3.3. LC‐MS/MS Quantification of Intra‐ and Extracellular Itaconate

APEC94 was added to macrophages at MOI = 10 and treated with gradient 4‐OI (100, 200, and 400 μM), metabolic inhibitors, tetracycline (1 μg/mL positive control), or DMSO/PBS vehicle at 2 h postinfection. At 0, 4, 8, 12, 16, 20, and 24 h, the cell culture supernatant (extracellular) and cell lysate (intracellular) were both collected. LC‐MS/MS was used to detect itaconate concentrations, which were normalized to the number of cells (nmol/10^6^ cells) and to the volume of the supernatant (mM).

##### 2.2.3.4. MTT Cell Viability Assay

The intervention reagents used for RAW264.7 cells were the same as described in Section 2.2.3.3, with the exception that the cells were treated for 24 h. A 5 mg/mL MTT working solution was added and incubated for 4 h at 37°C, and the OD_570_ was read after dissolving the formazan precipitate in DMSO. Cell viability was determined as a percentage of the DMSO solvent‐control group.

##### 2.2.3.5. Anti‐Inflammatory Western Blot of Macrophages

Macrophages were infected with APEC94 (MOI = 10), then treated with 400 μM 4‐OI or 1 μg/mL tetracycline for 24 h; uninfected PBS blank groups were set as controls. Total cellular protein was obtained by RIPA lysis buffer with protease inhibitors, separated by SDS‐PAGE, and transferred to PVDF after determination of 30 μg of protein per lane. Overnight incubation at 4°C with primary antibodies against the following: TNF‐α, IL‐1β, and GAPDH (as an internal reference) were followed by incubation with the HRP secondary antibody. The protein bands were then detected using ECL luminescence, and the relative expression was quantified using ImageJ software [22, 23].

#### 2.2.4. Quantitative Real‐Time PCR (qPCR) for Virulence Gene Transcription

APEC94 was treated with 400 μM 4‐OI or DMSO for 12 h; total RNA was extracted with Qiagen kits, and 1 μg of RNA was reverse‐transcribed into cDNA. The target virulence genes included *fimC* (adhesion), *luxS* (quorum sensing), *iroN* (iron uptake), *wza* (capsule synthesis), and *menB* (menaquinone synthesis), while 16S rRNA was used as an internal reference gene. Table 1 shows the sequences of the primers. qPCR thermal‐cycling profile: initial denaturation at 95°C for 3 min; 40 cycles of 95°C for 10 s, 58°C annealing for 30 s, and 72°C extension for 30 s; and final melt‐curve ramp from 65–95°C to verify amplicon specificity. Relative gene expression was calculated using the 2^−ΔΔCt^ method with 16S rRNA as an internal reference [17].

**Table 1:** Primer used in for RT‐qPCR.

Primer	Primer sequence (5′–3′)	Gene	Size (bp)
RT‐fimC F RT‐fimC R	CGAAAGCGCGGTTAAATTGC CCATTACGCCCGTCATTTTGG	*fimC*	127
RT‐menB F RT‐menB R	ACATCGGCGTGATCATTCTG AATCACCACGCACTTTCTGG	*menB*	78
RT‐wza‐F RT‐wza‐R	AGCGGCATCTTTGTCATTCG TAAGGTTGCAGGCGGAATTC	*wza*	131
RT‐iroN F RT‐iroN R	ATACACCAACCAGCCGGAAA TTCAGGTTGCCGTACAGACG	*iroN*	114
RT‐luxS F RT‐luxS R	GGATCCATACCCTGGAGCAC GCCCATTGGCGAGATATCGA	*luxS*	92
16S rRNA RT‐F 16S rRNA RT‐R	GCAAGCGTTATCCGGAATTG TCTACGCATTTCACCGCTAC	*16S rRNA*	150

#### 2.2.5. Molecular Mechanism Characterization

##### 2.2.5.1. NEM Fluorescence Detection of Free Cysteine Alkylation

After 12 h of incubation at 37°C, 400 μM 4‐OI‐ or DMSO‐treated cells of mid‐log APEC94 were collected by centrifugation (6000 × *g*, 10 min, 4°C), washed with precooled PBS, and subjected to lysis in protease‐containing RIPA buffer. The protein concentration in the supernatant was measured using the BCA assay. A total protein of 10 μg was added to 1 mM NEM reagent and incubated in the dark at room temperature for 30 min. Fluorescence values (Ex = 485 nm and Em = 520 nm) were measured using a microplate reader, with the DMSO control as 100%; the relative free thiol content of the 4‐OI group was calculated. The experiments were repeated three times as independent biological replicates.

##### 2.2.5.2. Biotin‐4‐OI Pull‐Down Combined With LC‐MS/MS Global Target Screening

APEC94 whole bacterial protein lysate (2 mg total protein) was incubated with 400 μM of 4‐OI or blank (DMSO) for 2 h at 37°C. Bound proteins were eluted from beads by SDS loading buffer at 95°C, separated by 12% SDS‐PAGE, followed by silver nitrate staining, and differential bands were cut for trypsin digestion and detected by LC‐MS/MS. The APEC94 protein database was searched against the mass spectrum data using the MaxQuant software, and proteins with ≥ 2 unique peptides detected only in the biotin‐4‐OI group were considered as candidate binding targets.

##### 2.2.5.3. Competitive Pull‐Down for Recombinant LuxS/Icl/OmpA

The purified recombinant LuxS, Icl, or OmpA protein (10 μg) was incubated with 10 μM biotin‐4‐OI at 37°C for 1 h. The 100‐fold excess unlabeled free 4‐OI was preincubated for 30 min before biotin‐4‐OI was added to the competitive groups. Binding complexes were captured with streptavidin agarose beads, washed, and subjected to boiling for the western blot detection of target proteins. Biological validation was performed via a competitive pull‐down assay: excess unlabeled native 4‐OI competed away probe binding to target proteins, confirming specific target engagement, as shown in Figure 1B.

**Figure 1:**
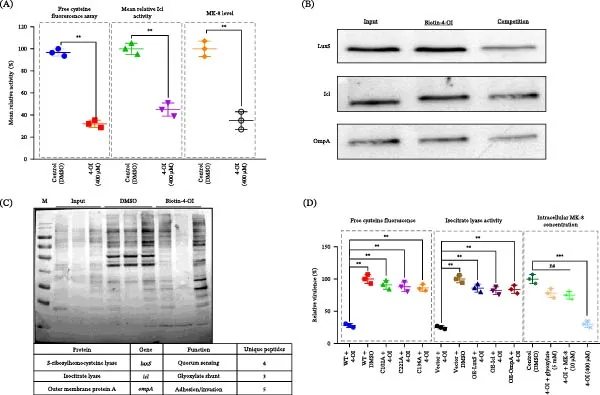
4‐OI directly targets LuxS, Icl, and OmpA via cysteine alkylation to attenuate APEC94 pathogenicity. (A) Free‐thiol fluorescence assay demonstrating reduction of total protein free‐thiol abundance in APEC94 whole‐cell lysates after 400 μM 4‐OI treatment. 4‐OI inhibits isocitrate lyase (Icl) activity, and also reduces intracellular menaquinone‐8 (MK‐8) levels. (B) Biotin‐4‐OI pull‐down and competition assay demonstrating specific binding of 4‐OI to LuxS, Icl, and OmpA. (C) SDS‐PAGE showing biotin‐4‐OI pull‐down replicates, with a table summarizing LC‐MS/MS‐identified targets. (D) Functional validation of identified targets. Site‐directed cysteine mutants (LuxS C102A, Icl C221A, and OmpA C136A) exhibited markedly reduced sensitivity to 4‐OI compared with wild‐type APEC94. Overexpression of LuxS, Icl, or OmpA partially restored bacterial virulence‐associated phenotypes during 4‐OI treatment. Supplementation with glyoxylate or menaquinone‐8 (MK‐8) also partially rescued bacterial function, supporting disruption of glyoxylate metabolism and menaquinone biosynthesis as downstream consequences of 4‐OI activity. Data are presented as mean ± SD (*n* = 3);  ^∗∗^ *p* < 0.01,  ^∗∗∗^ *p* < 0.001.

##### 2.2.5.4. Icl Enzymatic Activity Detection

APEC94 was treated with 400 μM 4‐OI or DMSO for 12 h, followed by lysis of the bacterial precipitate in precooled tris buffer (pH 7.5, 1 mM EDTA). The amount of protein in the supernatant was determined using the BCA method. The reaction system consisted of 50 mM Tris, 5 mM MgCl_2_, 2 mM DTT, 0.2 mM NADH, 2 U malate dehydrogenase, and 100 μg bacterial protein lysate in a 1 mL reaction system, which was started by the addition of sodium isocitrate. The decrease in OD_340_ was recorded in 10 min to determine the Icl activity using NADH molar extinction in 10 min. A value of 100% for the DMSO control was used.

##### 2.2.5.5. LC‐MS/MS Quantification of Intracellular Menaquinone‐8 (MK‐8)

APEC94 bacterial pellets were formulated as 4‐OI‐treated and control and resuspended in methanol, ice‐sonicated, and n‐hexane‐extracted. The organic phase was then dried with nitrogen and redissolved in methanol for positive ion MR mode (m/z 717.5 → 187.1) C1 column LC‐MS/MS detection. The standard curve was calculated for MK‐8, which was normalized to the DMSO control group, to determine the intracellular content.

##### 2.2.5.6. Metabolite Complementation Rescue Assay

Mid‐log APEC94 was treated with 400 μM 4‐OI and rescue groups with 5 mM sodium glyoxylate or 10 μM MK‐8, respectively, simultaneously, and DMSO as a blank. Macrophage adhesion and invasion assays for the detection of the “recovery” degree of the bacterial virulence were performed after 12 h of culture, using the blank control virulence as 100%.

##### 2.2.5.7. Construction of Δ*luxS*, Δ*icl*, Δ*ompA* Knockout and Complemented Strains

The λ‐red homologous recombination system (pKD46, pKD3, and pCP20 plasmids) was used to construct marker‐free single‐gene knockout strains. In brief, a chloramphenicol resistance cassette with 500‐bp homologous arms of the target genes was amplified by PCR and electroporated into pKD46‐competent APEC94 induced by L‐arabinose. PCR and sequencing were used to confirm the absence of resistance markers by digesting pCP20 at 42°C, and the positive clones were screened by chloramphenicol resistance. The full‐length coding sequences for *luxS*, *icl*, and *ompA* were cloned into the pBAD30 vector and introduced into the corresponding knockout strains after induction with 0.2% L‐arabinose. All of the bacterial phenotypic assays and macrophage infection assays were conducted on wild‐type, knockout, and complement strains with and without 400 μM 4‐OI to identify differences in bacterial virulence and sensitivity to 4‐OI.

##### 2.2.5.8. Cysteine‐to‐Alanine (C→A) Site‐Directed Mutagenesis

Cysteine residues in LuxS (C102), Icl (C221), and OmpA (C136) were changed to alanine with commercial point mutation kits based on the LC‐MS/MS analysis of the cysteine residues and the prediction of the protein structure. Mutated sequences were confirmed by DNA sequencing, and mutant proteins were expressed in the respective knockout strains. NEM fluorescence and biotin pull‐down assays were performed to determine if 4‐OI binds mutant proteins, and phenotypic assays were utilized to determine if 4‐OI lost inhibitory activity against mutant strains.

#### 2.2.6. In Vivo Murine Systemic Infection Assays

##### 2.2.6.1. Mouse Mortality and Tissue Colonization Assays

For survival monitoring, mice were injected intraperitoneally (i.p.) with 1 × 10^6^ CFU APEC94, and survival was recorded daily over 7 consecutive days. For tissue colonization detection, mice were infected with 1 × 10^8^ CFU of APEC94 and euthanized by CO_2_ inhalation at 24 h postinfection. Two distinct challenge doses were used: 1 × 10^6^ CFU for 7‐day survival monitoring (calibrated to yield interpretable partial mortality in vehicle‐infected controls). A higher 1 × 10^8^ CFU inoculum was used for 24 h tissue bacterial‐load quantification to ensure sufficiently high recoverable CFU across multiple tissue compartments for robust enumeration. A full summary of murine infection experimental parameters is provided in Supporting Information 1: Table S1. Serum, lung, liver, spleen, ileum, and cecum tissues were collected, homogenized in PBS containing 0.1% DOC, gradient diluted, and spread on LB plates to count tissue bacterial loads.

##### 2.2.6.2. Multiplex Cytokine Quantification

Tissue homogenate supernatant and mouse serum were extracted, and IL‐1β and TNF‐α concentrations were detected by the Luminex MAGPIX platform using a 32‐plex magnetic bead kit according to the manufacturer instructions [18]. Standard curves were used to calculate cytokine concentrations in each sample [24].

##### 2.2.6.3. In Vivo 4‐OI Dose Gradient Optimization

The APEC94‐infected mice were injected with 25, 50, and 100 mg/kg 4‐OI via an intraperitoneal injection, respectively. Serum TNF‐α levels were detected 24 h after treatment, and macrophage viability was measured by MTT to screen the optimal therapeutic dose.

#### 2.2.7. SPF Chicken Embryo Infection Model

A total of 45 (10‐day‐old) SPF embryos were randomly divided into three groups (*n* = 15). The groups consist of PBS + APEC94 control, low‐dose 4‐OI (25 mg/kg), and high‐dose 4‐OI (50 mg/kg). 4‐OI was injected into the allantoic cavity 2 h before the bacterial challenge (100 μL injection volume), followed by a 50 μL allantoic cavity injection of 1 × 10^6^ CFU of APEC94. Embryo survival was counted daily for 5 days; allantoic fluid was collected from surviving embryos on day 5 for CFU quantification.

#### 2.2.8. In Vivo Tissue Western Blot for LuxS/Icl/OmpA

Lung and liver tissues were homogenized in protease‐containing RIPA buffer, chicken embryo allantoic fluid was obtained, and total protein was extracted and quantified using BCA. The proteins (30 μg per lane) were separated in SDS‐PAGE and transferred to PVDF membranes. Primary antibodies: anti‐LuxS (1:1000), anti‐Icl (1:1000), anti‐OmpA (1:2000), and anti‐GAPDH (1:5000). Following secondary antibody incubation, protein bands were detected by ECL luminescence, and the relative protein abundance was quantified by ImageJ.

#### 2.2.9. Broad‐Spectrum Antivirulence Test of 4‐OI

Epidemic APEC serotypes (O1, O2, O18, O78, O88, and O145) and clinical multidrug‐resistant strains (resistant to ≥ 3 classes of antibiotics) were selected. There were 8–10 independent field isolates/reference strains for each serotype. Each strain was treated with 200–400 μM 4‐OI and then subjected to growth curve, biofilm assay, and macrophage adhesion to assess the broad‐spectrum inhibitory activity of 4‐OI against various APEC subtypes.

#### 2.2.10. Chicken Cecal Microbiota 16S rRNA Sequencing

The 10‐day‐old SPF embryos were injected with 4‐OI (50 mg/kg) or PBS control. Cecal contents were retrieved after 7 days of age after hatching. Microbial total DNA was extracted, and the hypervariable region of the 16S rRNA gene V3‐V4 was amplified for sequencing on the Illumina MiSeq platform. Shannon and Chao1 α‐diversity comparisons between groups were performed using bioinformatic analysis.

#### 2.2.11. Statistical Analysis

Each independent biological experiment was repeated at least three times, with technical replicates for each experiment. All quantitative data are presented as the mean ± standard error of the mean (SEM). GraphPad Prism 9 statistical software was used. One‐way ANOVA with the Tukey post hoc test was used for multiple group comparisons, while for mouse tissue colonization CFU data, the nonparametric Mann–Whitney *U* test was used. Significance thresholds:  ^∗^ *p*  < 0.05,  ^∗∗^ *p*  < 0.01,  ^∗∗∗^ *p*  < 0.001; ns = *p*  > 0.05 (no significant difference).

## 3. Results

We systematically characterized the antipathogenic mechanism of 4‐OI against APEC94 via layered in vitro phenotypic, ex vivo macrophage, biochemical genetic, and dual avian‐relevant in vivo models, sequentially uncovering phenotypic suppression, transcriptional virulence repression, direct cysteine alkylation targets, in vivo therapeutic efficacy, and poultry application safety.

### 3.1. 4‐OI Suppresses APEC94 Growth and Boosts Sensitivity to Osmotic and Acid–Base Stress

To first characterize the baseline inhibitory effects of 4‐OI on APEC94 fitness, we measured growth curves and stress resistance under 400 μM 4‐OI treatment, the optimal concentration validated by prior dose screening. Over a 24 h cultivation period, 4‐OI significantly slowed APEC94 proliferation, with persistently lower OD_600_ readings across all time points relative to the DMSO vehicle control (Figure 2A). For osmotic stress, increasing NaCl concentrations (0.5%–4% w/v) drastically reduced viable APEC94 CFU counts in the 4‐OI group, with maximal suppression observed at high salt gradients (Figure 2B). For physiological pH gradients spanning pH 4–10, 4‐OI selectively impaired bacterial viability at pH 5–7, with no measurable inhibitory effect under alkaline conditions (Figure 2C).

**Figure 2:**
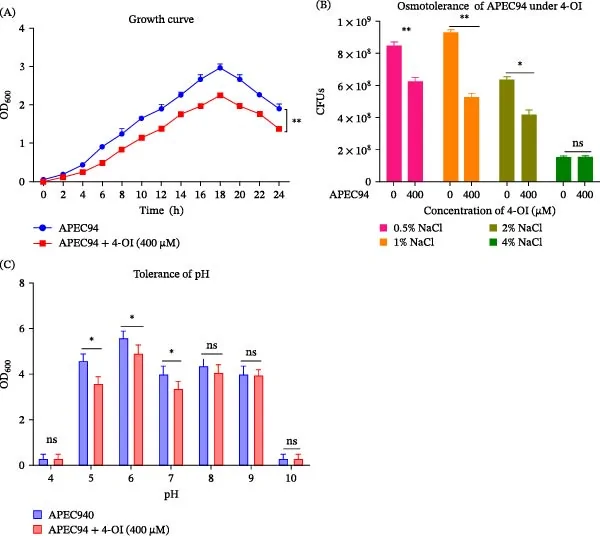
4‐OI disrupts core phenotypic traits critical for APEC94 pathogenicity. (A) Growth curves of APEC94 and APEC94 + 4‐OI strains in LB media. (B) Growth and survival of APEC94 and APEC94 + 4‐OI at different NaCl concentrations (osmotic stress). (C) Growth and survival of APEC94 and APEC94 + 4‐OI at different pH levels (acid–base stress). Data are presented as mean and SEM (*n* = 3). Statistical significance was determined by one‐way analysis of variance (ANOVA) followed by Tukey’s post hoc test ( ^∗∗^ *p* < 0.01,  ^∗^ *p* < 0.05, ns = *p* > 0.05), indicating differences between the indicated groups.

### 3.2. 4‐OI Abolishes Core Virulence Phenotypes

Intracellular persistence assays revealed that bacterial counts in RAW264.7 cells remained significantly lower in the 4‐OI‐treated group than in controls up to 16 h postinfection, confirming 4‐OI’s ability to suppress APEC94 survival within phagocytes (Figure 3A). Following this, 4‐OI further targeted virulence‐associated behaviors: biofilm formation was reduced by 64% (OD_595_) and swarming motility by 57% (mm) in 4‐OI‐treated APEC94 compared to that in controls (Figures 3B,C). Critically, 4‐OI impaired APEC94’s interaction with host cells by lowering the adhesion rate to 42% and the invasion rate to 35% in the 4‐OI‐treated group (Figures 3D,E). To partially disentangle growth‐rate‐dependent artifacts from specific antivirulence activity, key phenotypes were also assessed at subinhibitory 200 μM 4‐OI and normalized to viable bacterial counts (Supporting Information 2: Figure S3). Sub‐MIC 4‐OI still significantly attenuated virulence phenotypes independent of bulk‐growth suppression.

**Figure 3:**
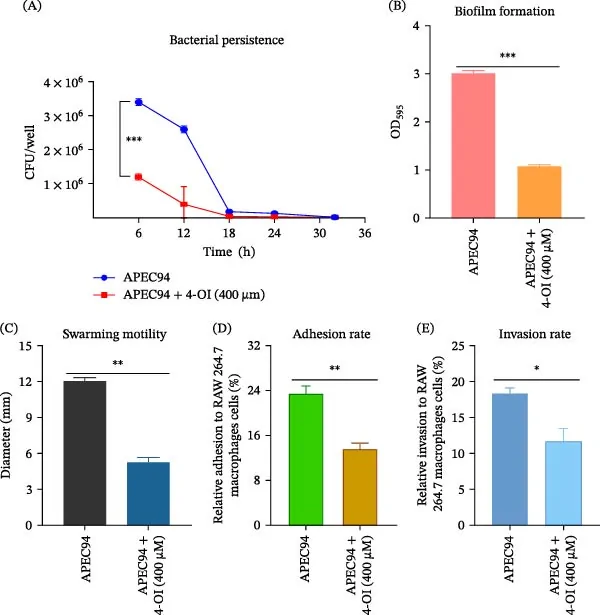
4‐OI abolishes core APEC94 virulence phenotypes required for host colonization and intracellular survival. (A) Time‐course intracellular APEC94 CFU in RAW264.7 macrophages over 6–36 h postinfection. (B) Crystal violet‐quantified biofilm formation. (C) Swarming motility diameter on 0.5% soft agar plates. (D) Macrophage adhesion CFU counts. (E) Macrophage invasive bacterial loads. Data = mean ± SEM, *n* = 3;  ^∗∗∗^ *p* < 0.001,  ^∗∗^ *p* < 0.01,  ^∗^ *p* < 0.05.

### 3.3. Dynamic Itaconate Secretion During APEC94 Macrophage Infection and 4‐OI‐Mediated Intracellular Bacterial Clearance

We profiled endogenous macrophage itaconate production during APEC94 infection to contextualize exogenous 4‐OI function. After bacterial challenge, extracellular itaconate accumulated time‐dependently, peaking at 0.18 mM at 24 h, while intracellular itaconate peaked at 16 h at 3.5 nmol/10^6^ cells before declining (Figure 4A,B). A dramatic shift in compartmentalization was observed: ~98% of itaconate resided intracellularly at 4 h, while >95% was exported extracellularly by 24 h (Figure 4C). These time‐course data demonstrate that endogenous macrophage‐synthesized itaconate undergoes pronounced compartment redistribution during APEC94 infection: it is predominantly retained intracellularly at early infection time‐points and is progressively exported to the extracellular milieu at later time‐points (>16 h postinfection). This temporal shift highlights the dynamic host‐metabolite response triggered by the APEC intracellular challenge.

**Figure 4:**
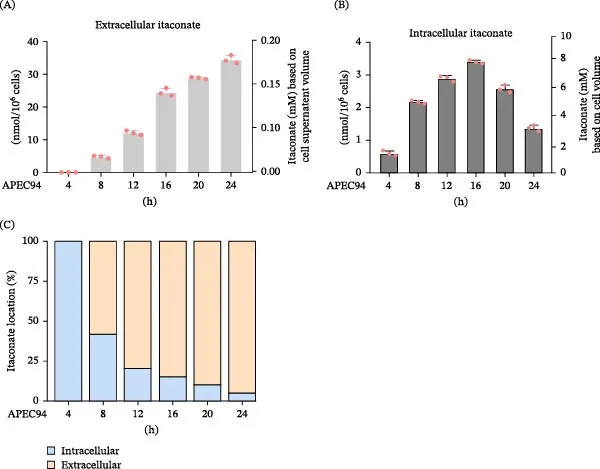
Endogenous itaconate compartment dynamics in RAW264.7 macrophages following APEC94 infection. (A) Extracellular itaconate concentrations in cell‐culture supernatants over 24 h (nmol/10^6^ cells). (B) Intracellular itaconate levels in RAW264.7 cells over 24 h (nmol/10^6^ cells). (C) Stacked bar chart showing the relative distribution of intracellular (blue) and extracellular (orange) itaconate over time. Data are presented as mean ± SEM (*n* = 3). Statistical significance was determined by ANOVA followed by Tukey’s post hoc test.

Dose gradient testing confirmed 400 μM 4‐OI achieves equivalent intracellular APEC94 clearance to 1 μg/mL tetracycline, whereas unrelated metabolic inhibitors (succinate, malonate, CCCP, etc.) failed to suppress intracellular bacterial loads (Figure 5A). Critically, 100–400 μM 4‐OI exerted no macrophage cytotoxicity (cell viability maintained at 90%–95%), unlike tetracycline, which significantly reduced host cell viability (Figure 5B).

**Figure 5:**
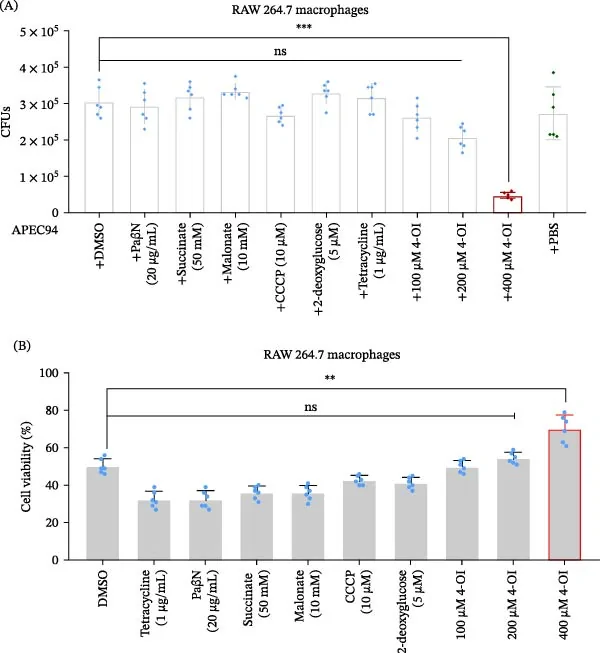
4‐OI clears intracellular APEC94 without macrophage cytotoxicity via a unique metabolic pathway. (A) Intracellular APEC94 CFU after treatment with gradient 4‐OI, metabolic modulators, tetracycline, or vehicle controls. (B) MTT‐determined RAW264.7 viability across all intervention groups. Data are presented as mean ± SEM (*n* = 6). Statistical significance was determined by ANOVA followed by Tukey’s post hoc test; ( ^∗∗^ *p* < 0.01,  ^∗∗∗^ *p* < 0.001, and ns = *p* > 0.05) indicate differences between the indicated groups.

### 3.4. 4‐OI Alleviates APEC94‐Induced Macrophage Inflammatory Overactivation

Uncontrolled proinflammatory cytokine release drives avian colibacillosis immunopathology, so we measured TNF‐α and IL‐1β protein abundance via western blot. APEC94 infection strongly upregulated both inflammatory mediators, while 400 μM 4‐OI treatment returned cytokine expression to baseline levels, matching tetracycline’s anti‐inflammatory effect (Figure 6A–C). Independent in vivo mouse dose trials identified 50 mg/kg as the optimal systemic therapeutic dose: (Figure 6D) 25, 50, and 100 mg/kg gradient administration reduced serum TNF‐α in a dose‐dependent manner, with no extra anti‐inflammatory benefit at 100 mg/kg (Figure 6E). Subsequent multiplex cytokine detection on mouse serum and intestinal tissues confirmed that 4‐OI robustly downregulated IL‐1β and TNF‐α in mucosal colonization sites (Supporting Information 2: Figure S1A,B). These findings demonstrate that 4‐OI effectively mitigates APEC94‐induced inflammatory injury while preserving the host cellular viability and therapeutic safety.

**Figure 6:**
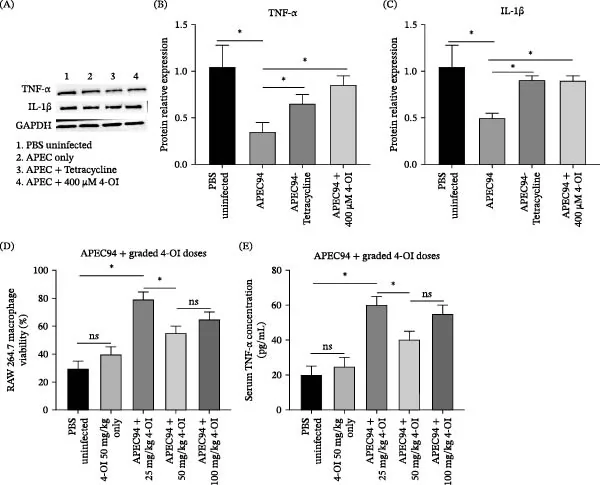
4‐OI relieves APEC94‐triggered macrophage inflammatory injury and defines optimal in vivo therapeutic dose. (A) WB protein expression of proinflammatory mediators TNF‐α and IL‐1β in RAW264.7 macrophages across treatment groups. (B) Relative TNF‐α protein expression levels in each group. (C) Relative IL‐1β protein expression levels in each group. (D) Macrophage viability assessment across graded in vivo 4‐OI doses in APEC94‐infected mice. (E) Assessment of 4‐OI dose‐dependent anti‐inflammatory activity in APEC94‐infected mice. Serum TNF‐α concentrations in APEC94‐infected mice treated with gradient doses of 4‐OI. Statistical analysis was performed using one‐way ANOVA followed by Tukey’s post hoc multiple comparison test;  ^∗^ *p* < 0.05; ns = nonsignificant.

### 3.5. 4‐OI Transcriptionally Represses Core APEC94 Virulence Genes

Dose–response phenotypic data confirmed maximal antivirulence activity at 400 μM in vitro; we next used qPCR to link phenotypic suppression to transcriptional repression of five master virulence genes selected from prior transcriptomics work. After 400 μM 4‐OI exposure, all target genes were significantly downregulated with fold repression values ranging from 2.4‐fold (*iroN*, iron uptake) to 5.3‐fold (*wza*, capsule synthesis); intermediate repression was observed for *fimC* (3.1×), *luxS* (3.7×), and *menB* (4.3×; Figure 7). These transcriptional changes provide a molecular basis for the global virulence attenuation observed in phenotypic assays [17]. These data link 4‐OI’s phenotypic effects to a broad downregulation of virulence‐associated pathways.

**Figure 7:**
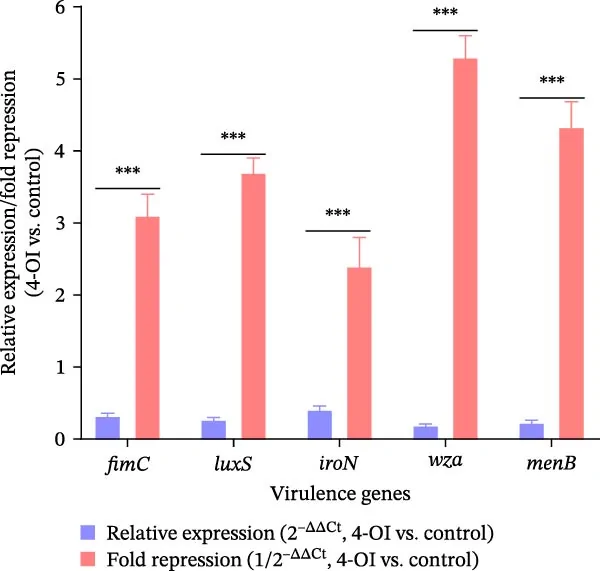
Transcriptional repression of core APEC94 virulence genes following 400 μM 4‐OI exposure. Relative expression (2^−ΔΔCt^, blue) and fold repression (1/2^−ΔΔCt^, red) of APEC94 virulence genes after 400 μM 4‐OI treatment, normalized to 16S rRNA. Data are presented as mean ± SEM (*n* = 3). Statistical significance was determined by one‐way ANOVA followed by Tukey’s post hoc test;  ^∗∗∗^ *p* < 0.001 indicates differences between the indicated groups.

### 3.6. 4‐OI Functions as a Cysteine Alkylator Targeting *luxS*, *icl*, and *ompA*

Having confirmed transcriptional repression of core virulence genes, we next performed a comprehensive panel of biochemical assays to identify the direct molecular targets of 4‐OI and validate its cysteine‐alkylation mechanism of action. NEM free‐thiol fluorescence assays revealed a marked reduction of free‐thiol groups in 4‐OI‐treated APEC94 lysates, demonstrating intracellular cysteine‐modifying activity. Functional metabolic readouts confirmed downstream pathway collapse: 4‐OI significantly suppressed Icl enzymatic activity and reduced intracellular MK‐8 concentrations (Figure 1A). Biotin‐4‐OI pull‐down assays verified specific physical interaction between 4‐OI and recombinant LuxS, Icl, and OmpA; excess unlabeled free 4‐OI fully competed off probe binding (Figure 1B). Mass spectrometry identified LuxS, Icl, and OmpA as high‐confidence interacting proteins with ≥ 2 unique peptides and strong spectral counts (Figure 1C). Exogenous supplementation with glyoxylate or MK‐8 partially restored bacterial virulence‐associated phenotypes, supporting that disruption of glyoxylate metabolism and menaquinone biosynthesis contributes to the antipathogenic activity of 4‐OI (Figure 1D). Genetic validation of the target requirement via gene knockout and cysteine site‐directed mutagenesis is presented in the following sections (Sections 3.7 and 3.8).

### 3.7. Gene Knockout Confirms *luxS*, *icl*, and *ompA* Are Indispensable for 4‐OI Antivirulence Activity

To genetically validate the three identified targets, we constructed single‐gene deletion mutants ΔluxS, Δicl, and ΔompA and their corresponding arabinose‐inducible complemented strains. Wild‐type APEC94 exhibited strong loss of biofilm, motility, adhesion, and intracellular survival under 4‐OI treatment; by contrast, all three knockout strains displayed severely blunted baseline virulence, with no further inhibitory effect after 4‐OI addition (Supporting Information 2: Figure S2A–D). Genetic complementation fully restored native virulence and reinstated 4‐OI sensitivity, demonstrating that *luxS*, *icl*, and *ompA* are mandatory molecular targets through which 4‐OI exerts antipathogenic effects.

### 3.8. Cysteine Residues Are Required for 4‐OI Binding and Functional Inhibition

We generated site‐directed C→A alanine mutants for the critical alkylation sites: LuxS(C102A), Icl(C221A), and OmpA(C136A). NEM fluorescence confirmed that 4‐OI no longer depletes free cysteine pools in mutant bacterial lysates (Figure 8A). Biotin pull‐down assays showed a complete loss of 4‐OI binding to mutant recombinant proteins (Figure 8B). Functional virulence assays demonstrated that cysteine‐mutant APEC94 strains were fully resistant to 4‐OI‐mediated suppression (Figure 8C), definitively establishing cysteine alkylation as the mandatory molecular mechanism of 4‐OI action.

**Figure 8:**
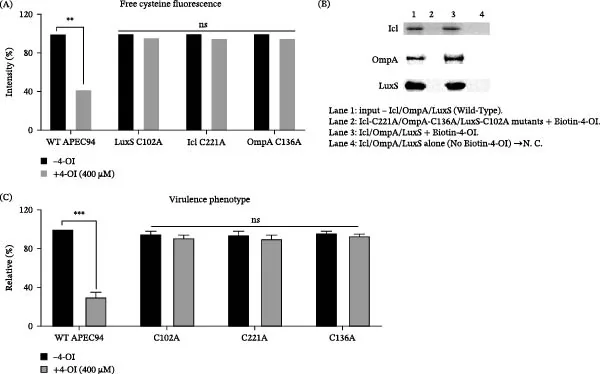
Cysteine point mutagenesis abolishes 4‐OI function. (A) Free thiol fluorescence of WT vs. cysteine‐mutant APEC94 with/without 4‐OI. (B) Pull‐down binding loss for mutant LuxS/Icl/OmpA. (C) Global virulence phenotype of cysteine mutant strains ±4‐OI. Mean ± SD, n = 3;  ^∗∗^ *p* < 0.01,  ^∗∗∗^ *p* < 0.001, ns = non significant.

### 3.9. In Vivo Murine Systemic Infection Model: 4‐OI Reduces Mortality and Tissue Colonization

We translated our in vitro mechanistic findings to a lethal systemic APEC94 mouse model using the optimized 50 mg/kg in vivo dose identified above. Untreated APEC94‐challenged mice reached 87.5% cumulative mortality over 7 days, while 4‐OI intervention cut mortality to just 12.5% (Figure 9A). Bacterial CFU quantification across systemic (serum, lung, liver, and spleen) and mucosal (ileum and cecum) tissues revealed drastically reduced APEC94 tissue burdens in the 4‐OI treatment group (Figure 9B–G). These in vivo data confirm that 4‐OI blocks APEC94 systemic dissemination and lethal pathogenicity in mammals.

**Figure 9:**
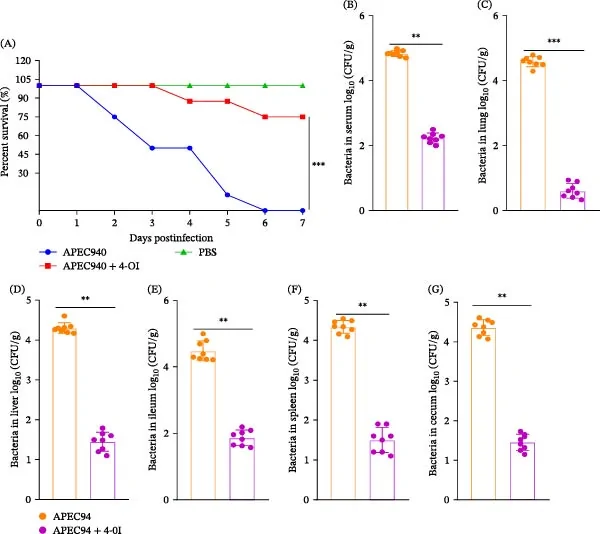
A 50 mg/kg 4‐OI mitigates APEC94 lethal infection and tissue colonization in C57BL/6 mice. (A) Seven‐day survival curve of APEC94‐infected mice with PBS/DMSO or 4‐OI treatment. (B–G) Recovered APEC94 CFU from serum, lung, liver, ileum, spleen, and cecum at 24 h postchallenge. *n* = 8 survival group; *n* = 3 tissue CFU replicates, mean ± SEM; Mann–Whitney *U* test,  ^∗∗^ *p* < 0.01,  ^∗∗∗^ *p* < 0.001.

### 3.10. In Vivo Target Protein Repression in Animal Tissues

Western blot analysis of the infected mouse lung and SPF chicken embryo allantoic fluid was performed to validate 4‐OI target suppression in living hosts. In mouse lung tissue, 4‐OI treatment reduced the LuxS protein abundance by 62%, Icl by 57%, and OmpA by 71% relative to infected untreated controls (Figure 10A). Consistent significant downregulation of all three target proteins was observed in chicken embryo allantoic fluid (Figure 10B), translating our in vitro cysteine alkylation mechanism into in vivo physiological conditions.

**Figure 10:**
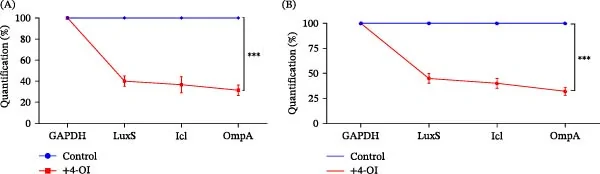
(A) In vivo western blot quantification of LuxS, Icl, and OmpA protein levels in mouse lung and (B) chicken embryo allantoic fluid from APEC94‐infected animals ±4‐OI. GAPDH is a loading control, mean ± SD, *n* = 3–5;  ^∗∗∗^ *p* < 0.001.

### 3.11. Dose‐Dependent Poultry‐Specific Therapeutic Efficacy

To evaluate the translational value for avian farming, we utilized 10‐day SPF chicken embryos, the gold‐standard avian infection surrogate. Two doses of 4‐OI were tested: a 25 mg/kg low dose and a 50 mg/kg optimized high dose (Figure 11). The PBS‐infected control group exhibited low embryo survival; 25 mg/kg 4‐OI raised the survival to 66.7%, while 50 mg/kg elevated the survival to 86.7%. Allantoic fluid bacterial loads were reduced by 61% (low dose) and 83% (high dose), confirming strong anti‐APEC94 activity in avian biological systems.

**Figure 11:**
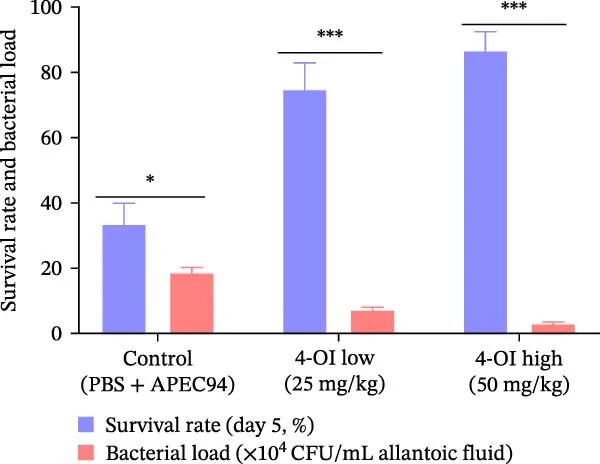
Five‐day survival rate and allantoic fluid APEC94 loads of APEC94‐challenged SPF embryos treated with PBS, 25 or 50 mg/kg 4‐OI, mean ± SEM, n = 15 embryos per group; one‐way ANOVA,  ^∗^ *p* < 0.05,  ^∗∗∗^ *p* < 0.001.

### 3.12. Broad‐Spectrum Activity Against Diverse APEC94 Serotypes and Multidrug‐Resistant Isolates

We tested 400 μM 4‐OI against six clinically dominant APEC serotypes (O1, O2, O18, O78, O88, and O145) and clinical MDR isolates resistant to ≥ 3 antibiotic classes. Uniformly robust suppression of biofilm formation was detected across all serotypes and MDR strains (Figure 12). Inhibitory potency was equivalent between wild‐type virulent strains and drug‐resistant isolates, proving that 4‐OI acts on conserved virulence/metabolic pathways unaffected by conventional antibiotic resistance mechanisms.

**Figure 12:**
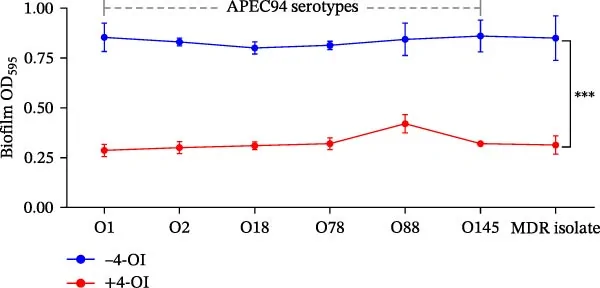
Biofilm inhibition rates of 4‐OI across major epidemic APEC94 serotypes and clinical MDR isolates, mean ± SD, *n* = 3;  ^∗∗∗^ *p* < 0.001 vs. untreated control strains.

### 3.13. 4‐OI Does Not Disrupt the Cecal Commensal Microbiota in Chickens

To evaluate the safety of embryonic 4‐OI administration for potential poultry application, 16S rRNA V3–V4 high‐throughput sequencing was conducted on cecal digesta collected from 7‐day‐old chicks subjected to in ovo injection of PBS vehicle or 50 mg/kg 4‐OI. We first compared the sequencing depth across experimental groups. The mean number of quality‐filtered sequencing reads did not differ significantly between PBS Control and 4‐OI treatment cohorts (Figure 13C), eliminating technical bias arising from uneven sequencing coverage. Subsequently, α‐diversity indices were analyzed to detect global shifts in the cecal microbial community structure. No significant intergroup differences were observed for the Chao1 richness index and Shannon diversity index (Figure 13A, B). The Chao1 richness index is a nonparametric estimator of community richness calculated based on the number of rare OTUs within each sample. The Shannon diversity index integrates both species richness and taxon relative abundance to describe community evenness. The comparable α‐diversity metrics between cohorts confirm that embryonic 4‐OI supplementation does not perturb cecal microbial complexity. These findings demonstrate that 4‐OI avoids commensal‐depleting gut dysbiosis frequently observed following exposure to broad‐spectrum antimicrobial interventions.

**Figure 13:**
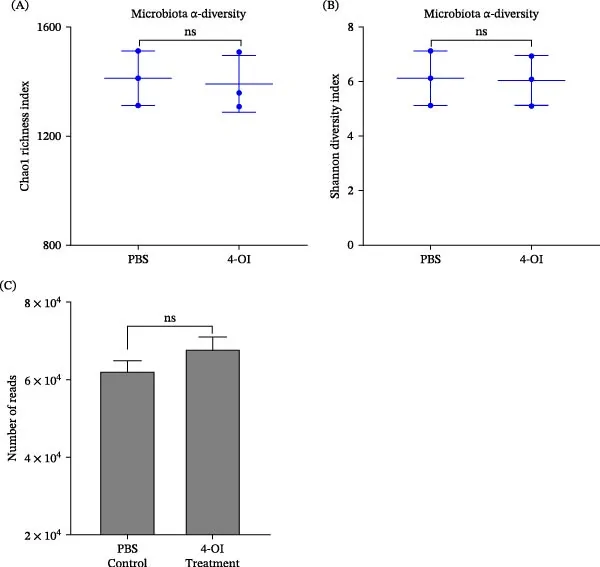
Cecal microbial α‐diversity profiles in chicks following embryonic administration. Box‐and‐scatter plot illustrating the Chao1 richness index (A), Shannon diversity index (B), and bar chart showing the number of quality‐filtered sequencing reads obtained from each sample (C) of PBS vehicle or 4‐OI treatment. Mean ± SD, *n* = 3; ns = nonsignificant.

## 4. Discussion

APEC is the primary causative agent of avian colibacillosis, triggering severe systemic inflammatory lesions including pericarditis, airsacculitis, and perihepatitis, causing up to 20% mortality in commercial broiler flocks and generating massive economic losses via reduced production performance and carcass waste [5, 25, 26]. More critically, widespread overuse of antibiotics has driven the rapid emergence of multidrug‐resistant APEC strains, creating severe zoonotic transmission risks to human food chains and severely limiting the clinical efficacy of conventional antibacterial drugs [7]. Current alternative prevention and control tools such as vaccines, probiotics, and bacteriophages all have obvious inherent defects: vaccines exhibit strict serotype restriction, probiotics rely on stable intestinal colonization capacity, and phages possess narrow host ranges with high mutation‐induced resistance risks [27]. Consistent with the three core research gaps raised in the introduction, there is an urgent unmet demand to develop broad‐spectrum, low‐resistance, safe antivirulence small molecules targeting APEC94’s core pathogenic pathways. Here, we systematically characterized the antipathogenic and immunomodulatory potential of 4‐OI, a cell‐permeable itaconate derivative, via complete layered in vitro, ex vivo, and dual poultry‐relevant in vivo experimental systems, and further decoded its unique cysteine alkylation molecular mechanism, filling critical blank areas in existing research.

Itaconate is a classic TCA cycle‐derived immunometabolite with dual antibacterial and anti‐inflammatory biological functions [8]. Native itaconate has strong polarity and poor membrane permeability, so it cannot efficiently penetrate bacterial and mammalian cell membranes, severely restricting its in vivo therapeutic application value [11, 15]. Ester‐modified 4‐OI retains the electrophilic thiol‐reactive structure of itaconate while greatly improving cell membrane permeability and can be metabolized to release bioactive itaconate intracellularly [18, 20]. Previous studies have confirmed that itaconate and its derivatives exert inhibitory effects on a series of pathogenic bacteria including *S. enterica*, *M. tuberculosis*, and *L. pneumophila*, mainly via indirectly inhibiting Icl, the rate‐limiting enzyme of the glyoxylate shunt that mediates pathogen survival in nutrient‐deficient host intracellular niches [11, 19, 28]. Partial studies also reported that 4‐OI inhibits biofilm and virulence gene expression in *Pseudomonas aeruginosa* and *Staphylococcus aureus*, but all existing literature ignores systematic research on APEC, and no prior work has identified direct bacterial protein binding partners or confirmed cysteine alkylation as the core antivirulence mode of action in Enterobacteriaceae pathogens [29]. Our laboratory’s preliminary transcriptomics work revealed widespread virulence gene downregulation after 4‐OI treatment of APEC94 but lacked phenotypic, cellular, and in vivo functional verification of these transcriptional changes [17, 30]. Based on this research background, this study constructed a complete experimental evidence chain to resolve the three major research gaps proposed in Section 1.

First, our in vitro phenotypic assays confirmed that 400 μM 4‐OI significantly suppresses the basic growth capacity of clinical O78‐type APEC94 and enhances its sensitivity to physiological osmotic and acid stress [5, 6]. At the pathogenic phenotype level, 4‐OI reduced biofilm formation by 64% and swarming motility by 57%, while lowering macrophage adhesion and invasion rates by 42% and 35%, respectively. Biofilm and motility are core prerequisites for APEC94 to colonize mucosal surfaces and spread in vivo; adhesion and invasion determine the efficiency of bacterial entry into immune cells to form persistent intracellular infection [17]. These phenotypic inhibitory effects provide direct macroscopic evidence that 4‐OI globally weakens APEC’s host colonization and immune evasion capacity, consistent with the core experimental expectation raised in our research hypothesis [31]. It should be noted that our primary in vitro virulence assays were performed at 400 μM 4‐OI, a near‐MIC bacteriostatic concentration that partially slows APEC94 proliferation. Reduced biofilm, motility, adhesion, and invasion measured at this concentration could theoretically arise in part from suppressed bacterial fitness rather than pure antivirulence activity. To address this confounder, we tested key phenotypes at sub‐MIC 200 μM 4‐OI and normalized readouts to viable recovered bacterial counts (Supporting Information 2: Figure S3). Significant virulence attenuation persisted under subinhibitory conditions, supporting that 4‐OI exerts specific antivirulence effects independent of bulk growth inhibition.

At the transcriptional regulatory level, qPCR data further explained the molecular basis of phenotypic attenuation: 4‐OI significantly downregulated five key virulence genes, *fimC*, *luxS*, *iroN*, *wza*, and *menB*, with repression folds ranging from 2.4 to 5.3 times [32]. Among them, *luxS* controls quorum sensing signal synthesis to regulate biofilm and motility; *wza* mediates capsular polysaccharide secretion to resist phagocytosis; *menB* participates in menaquinone synthesis to maintain bacterial respiratory chain function and oxidative stress tolerance; and *fimC* and *iroN* separately regulate fimbrial adhesion and iron uptake [13, 33]. This multi‐gene coordinated repression phenotype cannot be explained by simple indirect Icl inhibition reported in previous itaconate research, implying that 4‐OI possesses a broader multitarget regulatory mechanism in APEC, which prompted us to further explore its direct protein binding targets via biochemical and genetic means.

To decode the core molecular mechanism, we designed a complete set of cysteine alkylation detection, pull‐down screening, enzyme activity quantification, and gene editing verification experiments, which constitutes the most innovative part of this study different from all previous literature. NEM free thiol fluorescence assays first confirmed that 4‐OI significantly consumes free cysteine residues in APEC94 whole bacterial lysates, preliminarily verifying its cysteine modification activity [17]. Biotin‐labeled 4‐OI pull‐down combined with LC‐MS/MS unbiased proteomic screening identified three high‐confidence binding proteins, LuxS, Icl, and OmpA, and competitive pull‐down experiments using excess unlabeled 4‐OI further verified the specificity of this binding interaction [11].

Subsequent functional biochemical detection confirmed that 4‐OI strongly inhibits Icl enzymatic activity and reduces intracellular MK‐8 content; exogenous glyoxylate or MK‐8 metabolite supplementation can partially rescue APEC94 virulence defects, directly linking 4‐OI’s metabolic interference effect to its antipathogenic phenotype [17]. Most critically, single‐gene knockout (Δ*luxS*, Δ*icl*, and Δ*ompA*) and complement strain experiments proved that APEC94 loses virulence and no longer responds to 4‐OI inhibition after deleting any one of the three target proteins; C→A point mutation of the alkylation sites LuxS(C102), Icl(C221), and OmpA(C136) completely eliminates 4‐OI’s binding capacity and inhibitory activity [34].

At the ex vivo macrophage cellular level, this study simultaneously revealed dual antibacterial and anti‐inflammatory protective effects of 4‐OI. Endogenous itaconate produced by RAW264.7 macrophages show time‐dependent secretion characteristics after APEC infection, shifting from intracellular storage to extracellular release after 16 h, indicating that it acts as an endogenous antibacterial signal [18, 34]. Exogenous 4‐OI achieves equivalent intracellular APEC94 clearance efficiency to tetracycline at 400 μM but does not cause macrophage cytotoxicity, while tetracycline significantly damages immune cell activity [13]. Western blot detection of inflammatory proteins further showed that 4‐OI can significantly reduce APEC94‐induced TNF‐α and IL‐1β overexpression, alleviating excessive immune pathological injury triggered by infection [18]. Unlike most antibacterial drugs that only kill bacteria but cannot regulate host inflammation, 4‐OI’s dual‐function characteristic of simultaneously clearing intracellular pathogens and protecting immune cells highlights its unique therapeutic advantage for inflammatory infectious diseases such as avian colibacillosis.

Based on solid in vitro and cellular data, we further translated our findings into two poultry‐representative in vivo models to verify the translational therapeutic value. In the murine systemic lethal infection model, intraperitoneal injection of 50 mg/kg 4‐OI reduced APEC94‐induced mortality from 87.5% to 12.5% and significantly decreased bacterial loads in serum, lung, liver, spleen, and intestinal tissues, accompanied by systemic downregulation of proinflammatory cytokines IL‐1β and TNF‐α [15]. In 10‐day‐old SPF chicken embryo models, gradient dose screening confirmed 50 mg/kg as the optimal therapeutic dose: high‐dose 4‐OI raised embryo survival to 86.7% and reduced allantoic fluid bacterial load by 83%, while low dose (25 mg/kg) only achieved a 66.7% survival rate [16]. In vivo tissue western blot further validated that 4‐OI significantly downregulates LuxS, Icl, and OmpA protein abundance in infected mouse visceral tissues and chicken embryo allantoic fluid, proving the cysteine alkylation target inhibitory mechanism identified in vitro also functions in living animals, bridging the gap between the molecular mechanism and in vivo therapeutic effect [35].

To evaluate the industrial application potential of 4‐OI against diverse field APEC94 strains, we carried out broad‐spectrum activity tests covering six dominant epidemic serotypes (O1, O2, O18, O78, O88, and O145) and multiple clinical MDR isolates [36, 37]. A 400 μM 4‐OI uniformly inhibits biofilm formation and macrophage adhesion of all tested strains, with equivalent inhibitory efficiency against wild virulent strains and MDR isolates. This result is of great practical significance: conventional bactericidal antibiotics easily induce resistance by imposing strong survival screening pressure on bacteria, while 4‐OI only interferes with virulence rather than bacterial viability, targeting conserved metabolic and quorum‐sensing pathways shared by all APEC94 subtypes, so cross‐resistance with existing antibacterial agents will not occur [38, 39].

For feed additive safety evaluation, we conducted 16S rRNA V3‐V4 high‐throughput sequencing on chicken cecal microbiota. The results showed that the embryonic 50 mg/kg 4‐OI intervention does not cause significant changes in chicken cecal α‐diversity (Shannon and Chao1) or the relative abundance of dominant phyla Firmicutes, Bacteroidetes, and Proteobacteria [40]. Unlike antibiotics that severely disrupt intestinal commensal flora balance and impair broiler growth performance, 4‐OI’s neutral effect on poultry gut microbiota eliminates a key industrial application obstacle, providing complete safety data to support its development as a long‐term feeding additive for flocks [41]. Additionally, a comprehensive comparison between 4‐OI and existing APEC94 control strategies reveals three irreplaceable advantages for poultry breeding industry, i.e., low resistance selection risk, dual bacteria‐host regulation, and broad‐spectrum activity and flora safety [42]. From the formulation perspective, combined with our in vivo dose screening data, we propose two scalable delivery modes for commercial broiler production, i.e., encapsulated feed additives and hatchery aerosol sprays, to block early embryonic respiratory APEC94 colonization [43]. Important practical stability considerations apply for feed‐based delivery: the octyl‐ester moiety of 4‐OI is susceptible to hydrolytic breakdown under high‐temperature, high‐moisture feed‐extrusion processing. Lipid matrix microencapsulation will likely be required to preserve chemical integrity during commercial feed manufacturing; nonencapsulated 4‐OI may degrade under standard extrusion conditions. The optimal daily administration dose for growing broilers is predicted to be 40–60 mg/kg body weight, matching the effective concentration verified in chicken embryo models, and the physiological pH 5–7 of poultry digestive/respiratory tracts just fits 4‐OI’s optimal activity range [17, 44].

Limitations include the protective effect of 4‐OI needing further verification in commercial chicken feeding trials, as the SPF chicken embryo model used for avian efficacy evaluation cannot fully simulate the mature immune system and complex intestinal microecological environment of market‐age growing broilers. Further, this study only adopted single‐strain APEC94 infection models, while field poultry farms often face mixed infection of multiple APEC subtypes or co‐infection with *Salmonella* and other pathogens; the efficacy of 4‐OI under mixed bacterial pollution remains unknown [45]. Corresponding future research work includes constructing double/triple gene knockout strains of *luxS*, *icl*, and *ompA* to quantify the individual and synergistic contribution of each target to APEC virulence, performing global bacterial cysteine alkylation proteomics to screen additional potential 4‐OI binding proteins beyond the three targets identified in this work, and further clarifying the fine structural interaction mode between 4‐OI and cysteine residues via molecular docking and protein crystal analysis [40, 46]. Furthermore, the in ovo allantoic cavity administration route used in chicken embryo assays differs substantially from prospective oral feed additive delivery. Gastrointestinal luminal degradation, first‐pass host metabolism, and variable intestinal absorption will modify effective in vivo exposure under feeding conditions; these factors require dedicated assessment in follow‐on farm trials.

## 5. Conclusion

APEC‐mediated avian colibacillosis and the accompanying MDR crisis urgently demand safe alternative antipathogenic agents. In this study, we demonstrated that 4‐OI exerts global antivirulence effects on clinical APEC94 via covalent cysteine alkylation of three core functional proteins, LuxS, Icl, and OmpA, simultaneously inhibiting bacterial proliferation, stress tolerance, biofilm formation, and host cell invasion, while balancing macrophage inflammatory homeostasis without causing host cytotoxicity. In murine lethal infection and SPF chicken embryo avian models, 50 mg/kg 4‐OI achieves prominent protective efficacy, significantly reducing mortality and systemic bacterial colonization. 4‐OI maintains consistent inhibitory activity against all dominant APEC serotypes and clinical MDR isolates and does not disrupt chicken cecal commensal microbiota homeostasis. Gene knockout and cysteine point mutagenesis experiments definitively confirm that cysteine alkylation of *luxS*, *icl*, and *ompA* is the mandatory molecular mechanism of 4‐OI’s anti‐APEC function. Collectively, this study fills key research blanks in the mechanistic and translational research of itaconate derivatives against poultry pathogenic bacteria and provides a complete multi‐dimensional theoretical and experimental framework for developing 4‐OI as a low‐resistance, safe feed additive to combat avian colibacillosis and global livestock AMR.

## Author Contributions

**Saqib Nawaz**: writing – original draft, visualization, investigation, formal analysis, data curation. **Xiaoxian Liu and Haiyang Wang**: writing – review and editing, methodology, investigation. **Yaqin Tian, Jingwen Zhang, and Wei Jiang**: writing – review and editing, funding acquisition, conceptualization. **Xiangan Han and Song Xiangjun**: writing – review and editing, supervision, project administration, methodology, investigation, funding acquisition, conceptualization.

## Funding

We are thankful to the Chinese government for supporting us with funds from the National Natural Science Foundation of China (Grants U22A20518 and 32072829), Research Fund for International Scientists from the National Natural Science Foundation of China (Grant W2633101), and the Shanghai Agriculture Applied Technology Development Program (Grant T2023106).

## Disclosure

All authors have read and approved the final manuscript.

## Ethics Statement

All animal procedures complied with the Chinese national standard “Laboratory animal‐Guideline for ethical review of animal welfare” (GB/T 35892‐2018) and received institutional animal‐care‐and‐use‐committee ethical approval (Approval Number KJLLXM‐2026060) from Anhui Agricultural University.

## Conflicts of Interest

The authors declare no conflicts of interest.

## Supporting Information

Additional supporting information can be found online in the Supporting Information section.

## Supporting information


**Supporting Information 1** Table S1: Summary of murine in vivo infection experimental parameters. Table summarizes challenge dose, treatment groups, number of animals per group, total animal numbers, and experimental endpoints for three independent mouse infection experiments. CFU, colony‐forming units; i.p., intraperitoneal injection.

**Supporting Information 2** Figure S1: 4‐OI modulates inflammatory cytokine responses in APEC94‐infected mice. Quantification of (A) IL‐1β and (B) TNF‐α cytokine levels in serum, lung, liver, spleen, ileum, and cecum from PBS‐treated, APEC94‐infected, and APEC94‐infected + 4‐OI‐treated mice. Cytokine levels were quantified using the MILLIPLEX mouse cytokine/chemokine premixed 32‐plex magnetic‐bead panel. Data are presented as mean ± SD (*n* = 3 biological replicates). Statistical significance was determined by one‐way analysis of variance (ANOVA) followed by Tukey’s post hoc test ( ^∗^ *p* < 0.05,  ^∗∗^ *p* < 0.01,  ^∗∗∗^ *p* < 0.001). Figure S2: Phenotypic characterization of wild‐type APEC94, ΔluxS, Δicl, and ΔompA deletion mutants and their corresponding complemented strains with and without 400 μM 4‐OI treatment. (A) Quantification of biofilm formation (OD_595_). (B) Swarming motility (migration distance, mm). (C) Bacterial adhesion to macrophages (CFU/well). (D) Intracellular bacterial survival inside macrophages at 12 h postinfection (CFU/well). Data are presented as mean ± SD, *n* = 3 biological replicates.  ^∗^ *p* < 0.05,  ^∗∗^ *p* < 0.01; ns, nonsignificant. Figure S3: Sub‐MIC 200 μM 4‐OI suppresses APEC94 virulence phenotypes after normalization to viable bacterial CFU input. (A) Normalized biofilm biomass (crystal‐violet OD_595_ signal divided by recovered viable CFU). (B) Normalized swarming motility (swarm diameter normalized to recovered viable CFU). (C) Normalized macrophage adhesion (adhered CFU/input viable bacterial count). (D) Normalized macrophage invasion (invaded CFU/input viable bacterial count). Assays were performed at sub‐MIC 200 μM 4‐OI, well below the measured APEC94 MIC of 350 μM. All readouts are normalized to recovered viable CFU to correct for differences in bacterial growth. Data are presented as mean ± SEM (*n* = 3 biological replicates);  ^∗∗^ *p* < 0.01.

## Data Availability

The data that support the findings of this study are openly available in 4‐octyl itaconate attenuates APEC at https://data.mendeley.com/datasets/mt9nyrb9ck/1.
