# Supplementary material for: 4‐Octyl Itaconate Suppresses Virulence of Multidrug‐Resistant Avian Pathogenic Escherichia coli via Cysteine Alkylation of LuxS, Icl, and OmpA
Source: Transbound Emerg Dis. 2026 Oct 25;2026:5663447. doi: 10.1155/tbed/5663447 (PMC13695867; doi:10.1155/tbed/5663447)
Supplement: Supplementary file 1 [file TBED-2026-5663447-s001.docx]

**Supplementary Table 1. Summary of murine in vivo infection experimental parameters**

| **Experiment name** | **Challenge dose (I.P)** | **Treatment groups** | **n per group** | **Total animals** | **Experimental endpoint** |
| --- | --- | --- | --- | --- | --- |
| 7‑day survival monitoring | 1 × 10⁶ CFU APEC94 | 1. PBS uninfected control 2. APEC94 + DMSO vehicle 3. APEC94 + 50 mg/kg 4‑OI | n = 8 | 24 | Daily survival recording for 7 days post‑challenge; mortality assessment |
| 24‑h tissue bacterial load / organ colonization | 1 × 10⁸ CFU APEC94 | 1. PBS uninfected control 2. APEC94 + DMSO vehicle 3. APEC94 + 50 mg/kg 4‑OI | n = 8 | 24 | Euthanasia at 24 h post‑infection; CFU enumeration from serum, lung, liver, spleen, ileum, cecum; cytokine quantification in serum/tissue homogenates |
| In vivo 4‑OI dose‑gradient optimization | 1 × 10⁶ CFU APEC94 | 1. PBS uninfected control 2. APEC94 + DMSO vehicle 3. APEC94 + 25 mg/kg 4‑OI 4. APEC94 + 50 mg/kg 4‑OI 5. APEC94 + 100 mg/kg 4‑OI | n = 6 | 30 | 24 h post‑challenge; serum TNF‑α measurement, macrophage viability assay |

*i.p., intraperitoneal injection; CFU, colony‑forming units.
