# Supplementary material for: 4‐Octyl Itaconate Suppresses Virulence of Multidrug‐Resistant Avian Pathogenic Escherichia coli via Cysteine Alkylation of LuxS, Icl, and OmpA
Source: Transbound Emerg Dis. 2026 Oct 25;2026:5663447. doi: 10.1155/tbed/5663447 (PMC13695867; doi:10.1155/tbed/5663447)
Supplement: Supplementary file 2 [file TBED-2026-5663447-s002.docx]

**A**

**B**

**SUPPLEMENTARY FIGURE 1**: 4-OI modulates inflammatory cytokine responses in APEC94-infected mice. Quantification of (A) IL-1β and (B) TNF-α cytokine levels in serum, lung, liver, spleen, ileum, and cecum from PBS‑treated, APEC94-infected, and APEC94-infected + 4-OI-treated mice. Cytokine levels were quantified using the MILLIPLEX mouse cytokine/chemokine premixed 32-plex magnetic-bead panel. Data are presented as mean ± SD (n = 3 biological replicates). Statistical significance was determined by one-way analysis of variance (ANOVA) followed by Tukey’s post-hoc test (*p < 0.05, **p < 0.01, ***p < 0.001), indicating differences between the indicated groups.

**SUPPLEMENTARY FIGURE 2**: Phenotypic characterization of wild-type APEC94, ΔluxS, Δicl, ΔompA deletion mutants and their corresponding complemented strains with and without 400 μM 4-OI treatment. (A) Quantification of biofilm formation (OD₅₉₅); (B) Swarming motility (migration distance, mm); (C) Bacterial adhesion to macrophages (CFU/well); (D) Intracellular bacterial survival inside macrophages at 12 h post-infection (CFU/well). Data are presented as mean ± SD, n = 3 biological replicates. *p < 0.05, **p < 0.01; ns, non-significant.

**SUPPLEMENTARY FIGURE 3.** Sub-MIC 200 μM 4-OI suppresses APEC94 virulence phenotypes after normalization to viable bacterial CFU input. (A) Normalized biofilm biomass (crystal-violet OD₅₉₅ signal divided by recovered viable CFU). (B) Normalized swarming motility (swarm diameter normalized to recovered viable CFU). (C) Normalized macrophage adhesion (adhered CFU / input viable bacterial count). (D) Normalized macrophage invasion (invaded CFU / input viable bacterial count). Assays were performed at sub-MIC 200 μM 4-OI, well below the measured APEC94 MIC of 350 μM. All readouts are normalized to recovered viable CFU to correct for differences in bacterial growth. Data are presented as mean ± SEM (n = 3 biological replicates); **p < 0.01.
